# Enhanced Expression of IL-18 and IL-18BP in Plasma of Patients with Eczema: Altered Expression of IL-18BP and IL-18 Receptor on Mast Cells

**DOI:** 10.1155/2017/3090782

**Published:** 2017-08-03

**Authors:** Yalin Hu, Junling Wang, Huiyun Zhang, Hua Xie, Weiwei Song, Qijun Jiang, Nan Zhao, Shaoheng He

**Affiliations:** ^1^Allergy and Clinical Immunology Research Centre, The First Affiliated Hospital of Jinzhou Medical University, Jinzhou, Liaoning 121001, China; ^2^The PLA Center of Respiratory and Allergic Disease Diagnosing Management, General Hospital of Shenyang Military Area Command, Shenyang 110840, China; ^3^Department of Dermatology, Donggang Center Hospital, Donggang, Liaoning 118300, China

## Abstract

IL-18 has been found to be associated with eczema. However, little is known of the role of IL-18 binding protein (BP) and IL-18 receptor (R) in eczema. We therefore investigated the expression of IL-18, IL-18BP, and IL-18R on mast cells by using flow cytometry analysis and mouse eczema model. The results showed that plasma free IL-18 and free IL-18BP levels in eczema patients were higher than those in healthy controls. IL-18 provoked up to 3.1-fold increase in skin mast cells. IL-18 induced also an increase in IL-18BP+ mast cells, but a reduction of IL-18R+ mast cells in mouse eczema skin. It was found that house dust mite allergen Der p1 and egg allergen OVA induced upregulation of the expression of IL-18, IL-18BP, and IL-18R mRNAs in HMC-1 cells following 2 and 16 h incubation. In conclusion, correlation of IL-18 and IL-18BP in eczema plasma suggests an important balance between IL-18 and IL-18BP in eczema. The decrease in molar concentration ratio of plasma IL-18BP/IL-18 and allergen-induced upregulated expression of IL-18 and IL-18R in skin mast cells of the patients with eczema suggests that anti-IL-18 including IL-18BP therapy may be useful for the treatment of eczema.

## 1. Introduction

Eczema is a chronic condition of varying severity and may be the first manifestation of atopy [1]. It is associated with an immunoglobulin E- (IgE-) mediated response to food and allergens [2]. Although the pathogenesis of eczema remains obscure, the findings show that IL-18 in supernatants of PBMCs of patients with atopic eczema (AE) was significantly higher and that active IL-18 in sera of patients with AE was enhanced at the exacerbation of their disease [3] suggesting that IL-18 may play a role in eczema.

IL-18 is a potent proinflammatory cytokine involved in chronic inflammatory conditions such as cutaneous lupus erythematosus [4], psoriasis [5], and atopic dermatitis [6]. It is produced by skin resident dendritic cells [7] as well as by the most abundant cell type of upper skin layers, the keratinocytes [8], which is recognized as an important regulator of both innate [9] and acquired immunity [10].

The IL-18 receptor (IL-18R) is part of the IL-1R/TLR superfamily signaling via a MyD88-dependent pathway. A wide range of cells including Th1 cells [11], natural killer (NK) cells, natural killer T (NKT) cells, mast cells, and basophils express the IL-18R [12]. As for IL-18, IL-18R is also expressed in lesion skin of chronic inflammatory diseases such as psoriasis and cutaneous lupus erythematosus [13]. However, little is known of the expression of IL-18R in eczema.

IL-18 binding protein (BP) is an endogenous antagonist with high neutralising capacity that inhibits the action of IL-18 by preventing interaction with its cell surface receptors [14]. More precisely, it is noticed that a molar excess of 10 of IL-18BP over IL-18 is required to decrease a pathological level of 400 pg/ml of IL-18 to a level of a HC subject (40 pg/ml), and a molar excess of 20–25 of IL-18BP is needed to bring down free IL-18 to undetectable levels [15]. An imbalance between IL-18 and IL-18BP may account for increased IL-18 activity in eczema.

It is reported that increased number of mast cells was found in eczema skin [16], and the clinical severity of dermatitis correlated with the numbers of mast cells [17]. Mast cells generated from patients with atopic eczema have enhanced levels of granule mediators [18], and elevated levels of tryptase a mast cell unique product were observed in children with nummular eczema [19], indicating the involvement of mast cells in eczema. However, the expression levels of IL-18, its receptor IL-18R, and its antagonist IL-18BP in mast cells of eczema have not been investigated.

The aim of the current study is to investigate the correlation of IL-18 with IL-18BP in eczema, altered expression of IL-18, IL-18R, and IL-18BP in mast cells from eczema skin or cell line.

## 2. Materials and Methods

### 2.1. Reagent

The following reagents were purchased from Biolegend (San Diego, USA): BV421-conjugated mouse anti-human CD117, BV510-conjugated donkey anti-rabbit IgG polyclonal Ab, PE/Cy7-conjugated mouse anti-human CD34, PerCP/Cy5.5-conjugated mouse anti-human Fc*ε*RI*α*, PE-conjugated rat anti-mouse Fc*ε*RI*α*, PerCP/Cy5.5-conjugated rat anti-mouse CD117, Zombie Aqua™ Fixable Viability Kit, Zombie Green™ Fixable Viability Kit, human Fc receptor blocking solution, rat anti-mouse CD16/32, and all isotype antibodies. FITC-conjugated rabbit anti-mouse IL-18BP was from Cloud clone (Houston, USA). APC-conjugated mouse anti-human IL-18R*α*, PE-conjugated mouse anti-human IL-18, and APC-conjugated rat anti-mouse IL-18R were supplied by R&D Systems (Minneapolis, USA). Rabbit anti-human IL-18BP, goat anti-mouse IL-18BP, and biotin-conjugated goat anti-mouse IL-18BP were obtained from Abcam (Cambridge, UK). Group 1 house dust mite allergen Der p 1, calcium ionophore (CI), ovalbumin (OVA, grade V), trypan blue dye, collagenase, hyaluronidase, and DNase were purchased from Sigma-Aldrich (St Louis, MO, USA). Human IL-18BP and IL-18 ELISA kit were from ImmunoWay Biotechnology Company (CA, USA); Cytofix/Cytoperm™ Fixation/Permeabilization Kit was bought from BD Biosciences Pharmigen (Beldford, MA, USA). Fetal bovine serum (FBS, Hyclone) and RPMI 1640 were from Gibco BRL (Grand Island, NY, USA). Allergens for skin prick tests were supplied by ALK-Abelló, Inc. (Denmark). Most of the general purpose chemicals such as salts and buffer components were of analytical grade.

### 2.2. Subjects and Animals

A total of 31 patients with eczema and 15 healthy control (HC) subjects were recruited in the study. Their general characteristics were summarized in Table 1. The diagnosing criteria of eczema were conformed to the Schultz-Larsen criteria [20]. Skin tissues were obtained from children and adolescents with phimosis who were undergoing circumcision at the First Affiliated Hospital of Jinzhou Medical University, China. The suggested definition of phimosis from Dewan et al. [21] was adopted in this study. The informed consent from each volunteer according to the declaration of Helsinki and agreement with the ethical committee of the General Hospital of Shenyang Military Region of PLA and with the First Affiliated Hospital of Jinzhou Medical University was obtained.

Immediately after admission (acute exacerbation stage), the blood from each patient with eczema was collected. Blood from HCs were collected in the outpatient clinic. From each individual, 10 ml of peripheral blood was taken into an EDTA-containing tube before centrifugation at 450*g* for 10 min. The cells were used for flow cytometric analysis, and plasma was collected and frozen at −80°C until use.

BALB/c mice (4–6 w, 18–22 g) were obtained from Vital River Laboratory Animal Technology Co. Ltd. (Beijing, China), Certificate number 11400700118760. The animals were bred and reared under strict ethical conditions according to international recommendations. They were housed in the Animal Experimental Center of the First Affiliated Hospital of Jinzhou Medical University in a specific pathogen-free environment with free access to standard rodent chow and water, at a constant temperature 23–28°C and relative humidity of 60–75%. The animal experiment procedures were approved by the Animal Care Committee at Jinzhou Medical University.

### 2.3. Mouse Eczema Model

Mouse eczema model was mainly adopted from a previous study by Jin et al. [22] with modest modification. Briefly, female mice were anesthetized with ether and then shaved with a razor blade before stimulating with a dermaroller (soaked in 1.0 mg/ml OVA solution before contacting skin) for 5 times per day, which mimicked eczema skin lesion inflicted by scratching. Each mouse received the treatment every other day for 3 weeks. For control experiments, healthy mice received NS only instead of OVA solution. One week after the last stimulation, 50 *μ*l of 10 ng/ml of IL-18 or NS were injected subcutaneously around skin lesion area for 3 h before animals being sacrificed. Skin tissues of 3 × 3 cm^2^ around and far away from the injection site were removed for flow cytometric analysis.

### 2.4. Isolation of Tissue Cells and Flow Cytometric Analysis of IL-18, IL-18BP, and IL-18R Expression

The procedures for dispersing human and mouse skin tissue cells were mainly adopted from a previous study by He et al. [23]. Briefly, skin tissues were digested with collagenase, hyaluronidase, and DNase in RPMI 1640 medium supplemented with 5% (*v*/*v*) heat-inactivated FBS and 100 units/ml penicillin/streptomycin. After centrifugation, the cells were incubated with human Fc receptor blocking solution or anti-mouse CD16/32 and live/dead cell dyes (Zombie Aqua Fixable Viability Kit and Zombie Green Fixable Viability Kit for mouse [24] and human samples [25], resp.) for 15 min, for human samples, each labeled monoclonal antibody including PE/Cy7-conjugated anti-human CD34, PerCP/Cy5.5-conjugated anti-human FcεRI*α*, BV421-conjugated anti-human CD117, and APC-conjugated anti-human IL-18R were added into the tube; for mouse samples, PerCP/Cy5.5-conjugated anti-mouse CD117, PE-conjugated anti-mouse Fc*ε*RI*α*, and APC-conjugated anti-mouse IL-18R were added into the tube and incubated for another 15 min. After being washed with PBS, resuspended cells were fixed and permeabilized by using Cytofix/Cytoperm Fixation/Permeabilization Kit according to the manufacturer's instructions, and then PE-conjugated anti-human IL-18, BV510-conjugated anti-human IL-18BP, and FITC-conjugated anti-mouse IL-18BP were added for human and mouse sample, respectively. Finally, cells were washed with washing buffer and resuspended in fluorescence-activated cell sorting- (FACS-) flow solution and analyzed with FACS Verse flow cytometer (BD Biosciences, San Jose, CA). A total of 10,000 events in live cell gate were analyzed for each sample. Data were analyzed with FlowJo software version 7.0 (Treestar, Ashland, USA). Dead cells and doublets were excluded from analysis by live/dead cell dyes.

### 2.5. HMC-1 Cell Line Culture and Challenge

A human mast cell line, HMC-1, was a gift from Dr. Joseph H. Butterfield (Mayo Clinic, MN, USA). Cells were cultured in RPMI 1640 medium supplemented with 10% heat-inactivated FBS and 100 units/ml penicillin/streptomycin in 75 cm^2^ tissue culture flasks (Falcon) at 37°C in a 5% (*v*/*v*) CO_2_, water-saturated atmosphere.

The procedure for challenging HMC-1 cells was mainly adopted from a method previously described by He et al. for P815 cells [26]. Briefly, cultured HMC-1 cells at a density of 1 × 10^6^ cells/ml were incubated with Der p1 and OVA (both with concentrations of 0.03, 0.3, 1.0, and 3.0 μg/ml) for 30 min, 2 h and 16 h at 37°C, respectively, before being centrifuged at 450 ×g for 10 min at 4°C. Cell pellets containing approximately 1 × 10^6^ cells were resuspended in trizol for RT-PCR, and supernatant was collected and frozen at −80°C. The challenge tests were repeated 4 times.

### 2.6. RNA Isolation and qPCR Analysis

Total RNA was extracted from HMC-1 as described previously [27]. Briefly, after synthesizing cDNA from total RNA by using RT Master Mix Perfect Real Time, qPCR was performed with SYBR Premix Ex TaqII Kit on the ABI Prism 7700 Sequence Detection System (Perkin Elmer Applied Systems, Foster City, CA, USA). Each reaction contains 12.5 *μ*l of 2x SYBR green Master Mix, 300 nM oligonucleotide primers, and 10 *μ*l of the cDNA or plasmid DNA. Untreated controls were chosen as the reference samples, and the ΔCt for all experimental samples were subtracted by the ΔCt for the control samples (ΔΔCt). The magnitude change of test gene mRNA was expressed as 2^−ΔΔCt^. Each measurement of a sample was conducted in duplicate.

### 2.7. Determination of Levels of IL-18 and IL-18BP in Human Plasma and Culture Supernatant and Calculation of Molar Concentration Ratio of Plasma IL-18BP/IL-18

Levels of total IL-18 and IL-18BP in human plasma were detected by using ELISA kit according to the manufacturer's instructions. The calculation of free IL-18 and IL-18BP was based on a 1 : 1 stoichiometry in the complex of IL-18 and IL-18BPa with a dissociation constant (*KD*) of 0.4 nM [28]. The formula for calculation of molar concentration is that molar mass concentration divided by the relative molecular mass. The molar concentration ratio of plasma IL-18BP/IL-18 equals the molar concentration of free IL-18BP divided by the molar concentration of free IL-18.

### 2.8. Statistical Analysis

Statistical analyses were performed by using SPSS software (Version 17.0, IBM Corporation). Plasma levels of IL-18 and IL-18BP are presented as scatter plot. Data for the expression of IL-18, IL-18BP, and IL-18R in mast cells are displayed as a boxplot, which indicates the median, interquartile range, and the largest and smallest values for the number of experiments indicated. Where Kruskal-Wallis analysis indicated significant differences between groups, for the preplanned comparisons of interest, the paired Mann–Whitney *U* test was employed. Data for HMC-1 cell challenge study were expressed as mean ± SEM. Where analysis of variance indicated significant differences between groups with ANOVA, Student's *t*-test was applied. Correlations were determined by using Spearman rank correlation. For all analyses, *P* < 0.05 was taken as significant.

## 3. Results

### 3.1. Elevated Levels of IL-18 and IL-18BP in Eczema Plasma

**Figure 1:**
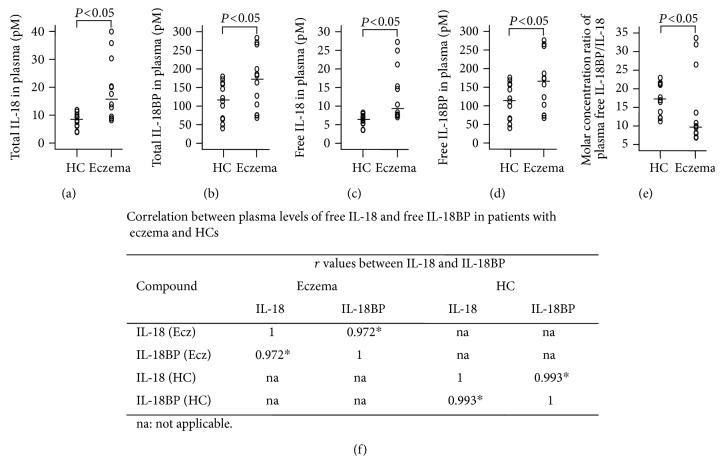
Scatter plots of levels of IL-18 and IL-18BP in plasma of patients with eczema and healthy control (HC) subjects. Levels of total IL-18 (a), total IL-18BP (b), free IL-18 (c), and free IL-18BP (d) and the molar concentration ratios of plasma IL-18BP/IL-18 (e) were shown. Each symbol represents the value from 1 subject. The median value is indicated with a horizontal line. *P* < 0.05 was taken as statistically significant. (f) The spearman's rho/correlation coefficient between the plasma level of free IL-18 and free IL-18BP. ^∗^*P* < 0.05.

It was observed previously that IL-18 in supernatants of PBMCs of patients with AE was significantly higher and that active IL-18 in sera of patients with AE was enhanced at the exacerbation of their disease [3]. However, the plasma/serum level of IL-18BP in eczema and correlation between IL-18 and IL-18BP have not been investigated. Using ELISA kits, we observed that levels of total IL-18 (Figure 1(a)), total IL-18BP (Figure 1(b)), calculated free IL-18 (Figure 1(c)), and calculated free IL-18BP (Figure 1(d)) in the plasma of patients with eczema were elevated in comparison with HC subjects. The top 3 highest levels of total IL-18 in patients with eczema were 30.3, 35.8, and 40.0 pM (Figure 1(a)), and the corresponding levels of total IL-18BP were 182.2, 185.5, and 199.6 pM (Figure 1(b)), respectively. The top 3 highest levels of free IL-18 in patients were 21.2, 25.0, and 27.2 pM (Figure 1(c)), and the corresponding levels of free IL-18BP were 173.0, 174.6, and 186.9 pM (Figure 1(d)), respectively. Molar concentration ratios of IL-18BP/IL-18 were 17.3 (11.2–23.0) and 9.93 (6.9–33.7) in plasma of HC subjects and patient with eczema, respectively (Figure 1(e)). It was observed that IL-18 and IL-18BP was correlated well with each other in the plasma of patients with eczema and HC subjects (Figure 1(f)).

### 3.2. Flow Cytometry Analysis of the Expression of IL-18, IL-18BP, and IL-18R in Human Skin Mast Cells

**Figure 2:**
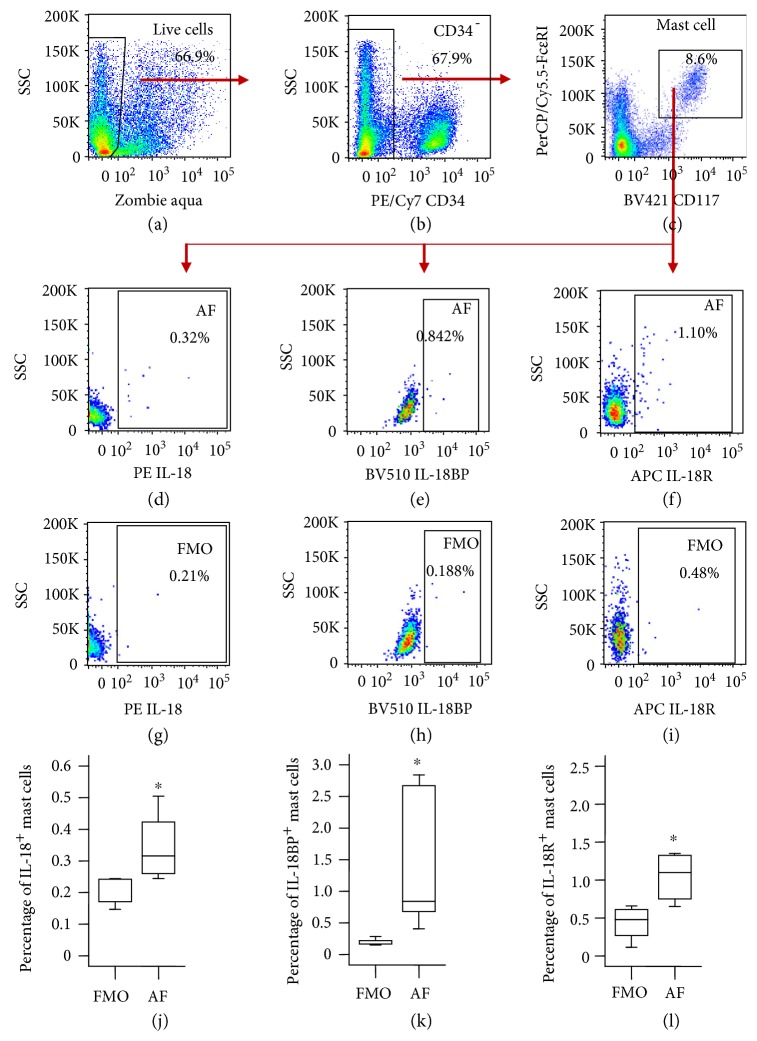
Expression of IL-18, IL-18 receptor (IL-18R), and IL-18 binding protein (BP) in mast cells from human skin tissue of healthy subjects. (a)–(c) show gating strategy of mast cells; (d)–(f) show percentage of IL-18, IL-18BP, and IL-18R expressing mast cells; (g)–(i) show fluorescence minus one (FMO) of IL-18, IL-18BP, and IL-18R expressing mast cells; and (j)–(l) are percentage values of IL-18, IL-18BP, and IL-18R expressing mast cells in skin tissue. Data shown are the median (range) value from 6 different donors. ^∗^*P* < 0.05 in comparison with FMO (paired Mann–Whitney *U* test). AF: all fluorescence.

It is reported that increased number of mast cells was found in eczema skin [16], and mast cells generated from patients with atopic eczema have enhanced levels of granule mediators [18]. However, little is known of the expression levels of IL-18, IL-18R, and IL-18BP in mast cells. Using flow cytometry technique, we investigated the expression of IL-18, IL-18R, and IL-18BP in dermal mast cells. The results showed that approximately 0.32, 0.8, and 1.1% skin mast cells express IL-18 (Figures 2(d) and 2(j)), IL-18 BP (Figures 2(e) and 2(k)), and IL-18R (Figures 2(f) and 2(l)), respectively, in normal human foreskin.

### 3.3. Increased Number of Mast Cells in Eczema Skin of Mice

**Figure 3:**
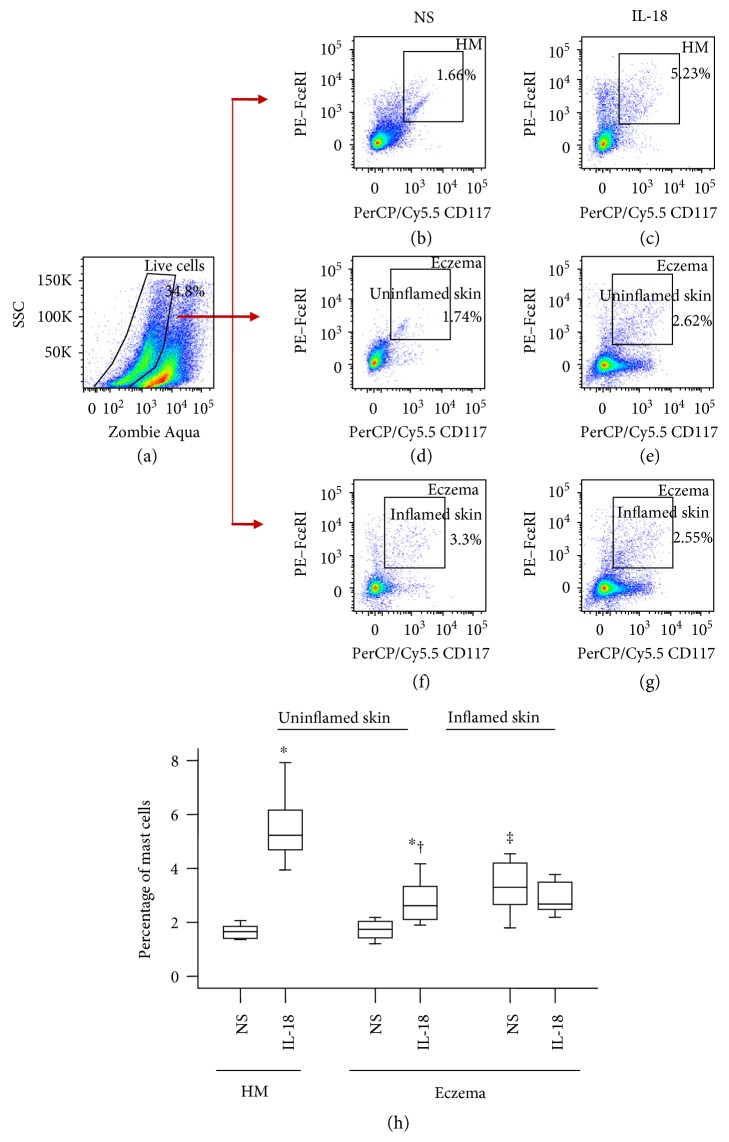
Flow cytometry analysis of the expression of IL-18 in dispersed skin mast cells of mice. Mice were treated with OVA solution for 3 weeks. For control experiments, healthy mice received NS only instead of OVA solution. After IL-18 or NS being injected subcutaneously around skin lesion area for 3 h, skin tissues of 3 × 3 cm^2^ around and far away from the injection site were removed and digested. Dispersed skin mast cells were then analyzed by flow cytometry. (a) represents gating strategy for live cells; (b), (d), and (f) show percentage of mast cells in skin of healthy mice (HM) and in normal or inflammatory skin of eczema mice treated with normal saline (NS), respectively. (c), (e), and (g) show percentage of mast cells in skin of healthy mice (HM) and in normal or inflammatory skin of eczema mice treated with IL-18 (10 ng/ml), respectively. (h) represents median percentage values of mast cells in mouse skin. Data shown are the median (range) value from 6 different mice. ^∗^*P* < 0.05 in comparison with NS group. ^†^*P* < 0.05 compared with HM IL-18 group. ^‡^*P* < 0.05 compared with HM NS group (paired Mann–Whitney *U* test).

In order to investigate role of mast cells in eczema, we developed a mouse eczema skin model. The results showed that mast cell numbers in inflamed area of mouse eczema skin (Figure 3(f)) were markedly greater than that in uninflamed area (Figure 3(d)) and in skin of healthy mice (Figure 3(b)). IL-18 at 10 ng/ml provoked 3.1 and 1.5-fold increase in skin mast cells of healthy mice and uninflamed area of eczema mice (Figure 3(h)).

### 3.4. Induction of Increased Number of IL-18BP+ Mast Cells in Eczema Skin of Mice by IL-18

**Figure 4:**
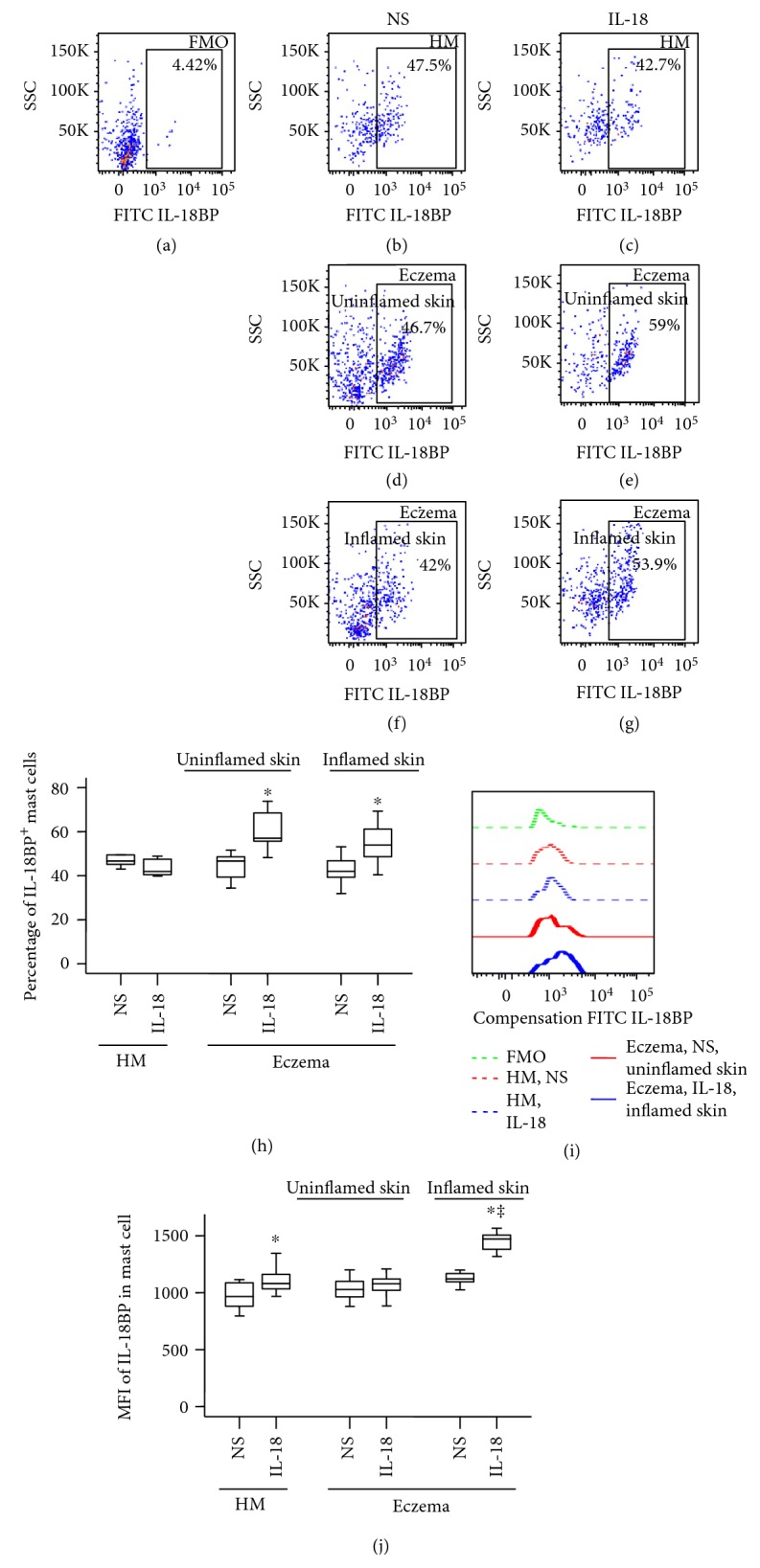
Flow cytometry analysis of the expression of IL-18 binding protein (BP) in dispersed skin mast cells of mice. (a) FMO for IL-18BP; (b), (d), and (f) show percentage of IL-18BP+ mast cells in skin of healthy mice (HM) and in normal or inflammatory skin of eczema mice treated with normal saline (NS), respectively. (c), (e), and (g) show percentage of IL-18BP+ mast cells in skin of healthy mice (HM) and in normal or inflammatory skin of eczema mice treated with IL-18, respectively. (h) represents median percentage values of mast cells in mouse skin. (i) shows representative flow cytometric figures of mean fluorescence intensity (MFI) of IL-18BP+ mast cell. (j) demonstrates changes in MFI of IL-18BP+ mast cell. Data shown are the median (range) value from 6 different mice. ^∗^*P* < 0.05 in comparison with NS group (paired Mann–Whitney *U* test). ^‡^*P* < 0.05 compared with HM NS group.

IL-18 BP is an endogenous antagonist with high neutralising capacity that inhibits the action of IL-18 by preventing interaction with its cell surface receptors [14]. An imbalance between IL-18 and IL-18BP may account for increased IL-18 activity in eczema. In order to understand the relationship between IL-18BP and mast cells, we examined the expression of IL-18BP in mast cells. The result showed that more than 40% mouse skin mast cells expressed IL-18BP regardless of allergen sensitization. IL-18 provocation increased percentage of IL-18BP+ mast cells in both inflamed (Figure 4(g)) and uninflamed skin areas (Figure 4(e)) of mouse eczema skin (Figure 4(h)) by 38.2% and 26.2% in comparison with that in healthy skin of mice (Figure 4(c)).

### 3.5. Induction of Decreased Number of IL-18R+ Mast Cells in Uninflamed Skin of Mice by IL-18

**Figure 5:**
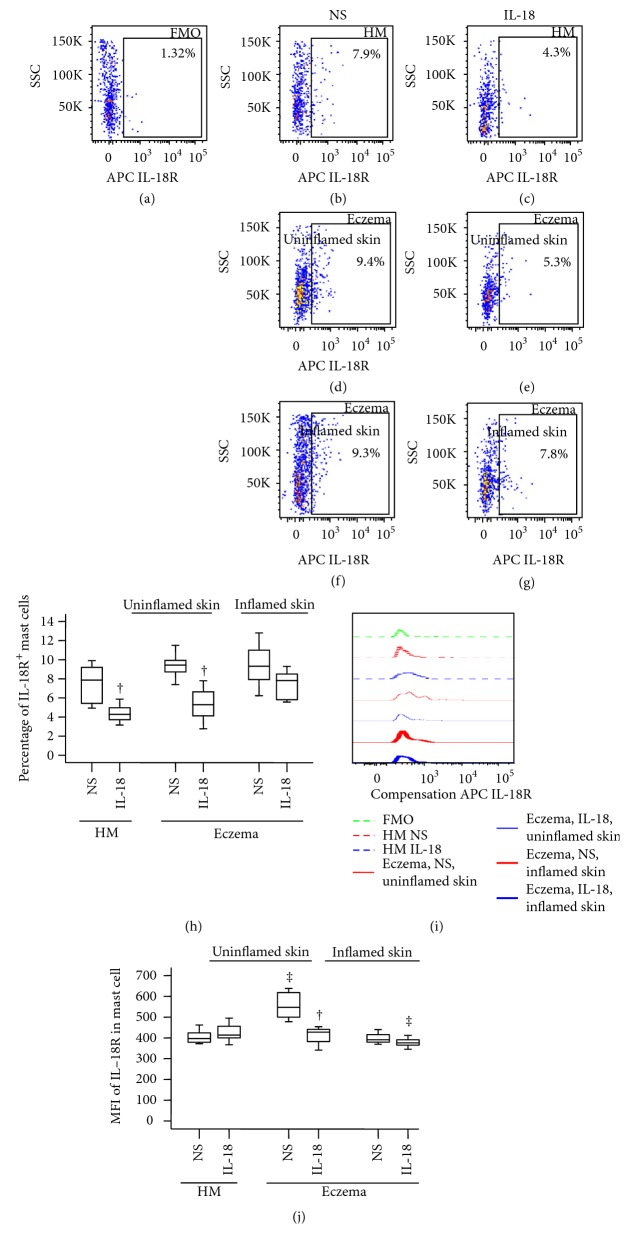
Flow cytometry analysis of expression of IL-18 receptor (IL-18R) in dispersed skin mast cells of mice. (a) FMO for IL-18R; (b), (d), and (f) show percentage of IL-18R+ mast cells in skin of healthy mice (HM) and in normal or inflammatory skin of eczema mice treated with normal saline (NS), respectively. (c), (e), and (g) show percentage of IL-18R+ mast cells in skin of healthy mice (HM) and in normal or inflammatory skin of eczema mice treated with IL-18, respectively. (h) represents median percentage values of mast cells in mouse skin. (i) shows representative flow cytometric figures of mean fluorescence intensity (MFI) of IL-18BP+ mast cell. (j) demonstrates changes in MFI of IL-18BP+ mast cell. Data shown are the median (range) value from 6 different mice. ^∗^*P* < 0.05 in comparison with NS group (paired Mann–Whitney *U* test) ^‡^*P* < 0.05 compared with HM NS group. ^†^*P* < 0.05 compared with corresponding NS group.

It has been noticed that mast cells express IL-18R [12]. However, little is known of the expression of IL-18R in eczema and influence of IL-18 on IL-18R expression on mast cells. To further evaluate the role of IL-18R in eczema, we examined the effect of IL-18 on IL-18R expression on mast cells of eczema mice. The results showed that IL-18 reduced the number of IL-18R+ mast cells in the skin of healthy mice (Figure 5(c)) and in uninflamed skin area of eczema mice (Figure 5(e)) by approximately 45.6% and 44.1%, respectively (Figure 5(h)).

### 3.6. Induction of Upregulated Expression of IL-18, IL-18BP, and IL-18R mRNA in HMC-1 Cells by Allergens

**Figure 6:**
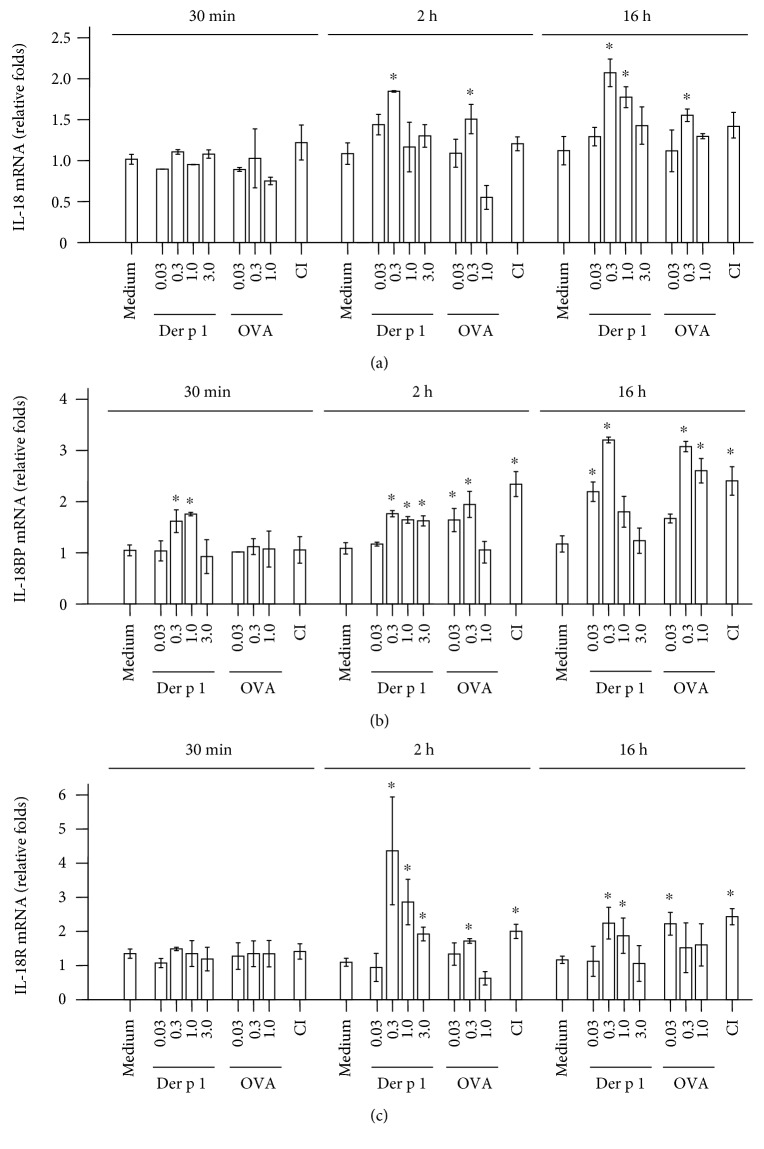
Quantitative real-time PCR (qPCR) analysis of the expression of IL-18, IL-18BP, and IL-18 receptor (IL-18R) mRNA in HMC-1 mast cells. Cells were challenged by various concentrations of group 1 house dust mite allergen Der P 1, ovalbumin (OVA), or calcium ionophore (CI) at 37°C for 2 h and 16 h. The expression of IL-18 (a), IL-18BP (b), and IL-18R (c) mRNAs were analyzed by qPCR. The data were expressed as mean ± SE for four separate experiments performed in duplicate. ^∗^*P* < 0.05 compared with the response to corresponding medium alone control. *P* < 0.05 in comparison of the reduced response with the response to the corresponding allergen alone. *P* < 0.05 in comparison of the enhanced response with the response to the corresponding allergen alone.

It was reported that house dust mite allergen Der p1 [29] and egg allergen OVA [30] are associated with the development of eczema. It is possible that these two allergens act on mast cells. We therefore examined the potential influence of Der p1 and OVA on the expression of IL-18, IL-18BP, and IL-18R mRNA in HMC-1 cells. It was found that Der p1 and OVA induced up to 84.9 and 38.7% increase in the expression of IL-18 mRNA over baseline control, respectively, at 16 h following incubation (Figure 6(a)). Similarly, Der p1 and OVA induced up to 1.7 and 1.6-fold increase in the expression of IL-18BP mRNA over baseline control, respectively, at 16 h following incubation (Figure 6(b)). Der p1 and OVA induced also up to 4.5 and 0.6-fold increase in the expression of IL-18R mRNA over baseline control, respectively, at 2 h following incubation (Figure 6(c)).

## 4. Discussion

We have demonstrated that levels of IL-18 and IL-18BP were elevated in the plasma of patients with eczema. We also found for the first time that the level of IL-18 correlated well with the level of IL-18BP in the plasma. Since active sera IL-18 was enhanced at the exacerbation of AE [3], IL-18 is a potent proinflammatory cytokine involved in several chronic skin inflammatory diseases [4–6], and IL-18BP is an endogenous antagonist with high neutralising capacity against IL-18 [14]; our finding emphasizes further the importance of IL-18 and balance between IL-18 and IL-18BP in the pathogenesis of eczema. Some previous reports demonstrated the importance of the balance between IL-18 and IL-18BP in inflammatory bowel disease [31], in rheumatoid arthritis [32], and in systemic lupus erythematosus [33] which may help to understand our finding that plasma level IL-18 correlated well with IL-18BP level in eczema.

IL-18 is a member of the IL-1 family and known to have potent proinflammatory effects by initiating an inflammatory cytokine cascade [34, 35]. It provoked the upregulation of the expression of MHC II molecules, production of chemokine CXCL10/IP-10 from keratinocytes [13], production of IL-4 and IL-13 by CD4^+^ T cells [36] and basophils [37], and production of IgE [38]. It has been noticed that IL-18 contributes to the spontaneous development of atopic dermatitis-like inflammatory skin lesion [39] and skews, the invariant NKT-cell population via autoreactive activation in AE [40]. These observations implicate that IL-18 may contribute to the development of eczema.

Although unlike in rheumatoid arthritis, that the expression of IL-18BP was decreased [32], it is observed that both plasma levels of IL-18 and IL-18BP in eczema were increased. Therefore, a molar concentration ratio of free plasma IL-18BP/IL-18 appears to be a key factor to determine the role of IL-18 in eczema. Indeed, it is reported that a molar excess of 10 of IL-18BP over IL-18 is required to decrease a pathological level of 400 pg/ml of IL-18 to a level of a HC subject (40 pg/ml) [15]. Since the current experiment demonstrated that the molar concentration ratio of free plasma IL-18BP/IL-18 was 9.93 in the patient with eczema, it seems likely not enough IL-18BP to inhibit proinflammatory actions of IL-18 in eczema. Therefore, the anti-IL-18 therapy may be useful for the treatment of eczema.

It is reported that increased number of mast cells in the lesion skin of eczema [41] correlated with the clinical severity of dermatitis in eczema skin, and mast cells generated from patients with atopic eczema have enhanced levels of granule mediators [18], indicating the involvement of mast cells in eczema. In order to determine the potential sources of IL-18 and IL-18BP, we examined the expression levels of IL-18, its receptor IL-18R, and its antagonist IL-18BP in human skin mast cells. The results showed that only small proportions of mast cells express IL-18 and IL-18BP. Considering mast cells only consist of approximately 4% dispersed skin cells [42], small proportions of IL-18+ and IL-18BP+ mast cells are hardly a major source of IL-18 and IL-18BP. Many other cell types including keratinocytes and dermal macrophages and dendritic cells are capable of producing IL-18 [43]; human keratinocytes and fibroblasts can secrete IL-18BP [44].

On the other hand, large proportion of mouse mast cells expresses IL-18BP regardless of allergen sensitization, indicating the marked difference of IL-18BP expression between the two species. Although it is not significant, eczema skin mast cells seem to produce less IL-18BP than HC skin mast cells, which supports the view that IL-18 play a role in eczema via reduced IL-18BP production in the body. Nevertheless, the ability of IL-18 in provocation of increased IL-18BP+ mast cells in both inflamed and uninflamed areas of mouse eczema skin suggests that enhanced plasma level of IL-18BP in eczema may be partially released from mast cells induced by IL-18. However, IL-18 seems to downregulate the expression of IL-18R on mouse mast cells from uninflamed tissue, suggesting that IL-18-induced elevation of IL-18BP+ mast cells may not be via IL-18R. It could result from an indirect mechanism such as through an interferon gamma- (IFN-*γ*-) dependent pathway, which IL-18 provokes an abundant IFN-*γ* release from human NK cells [45], released IFN-*γ*, and then induces IL-18BP [46, 47] release from mast cells through IFN-*γ* receptor expressing on mast cells [48]. Obviously, more work is required to address these issues. Induction of the upregulated expression of IL-18, IL-18BP, and IL-18R on human mast cells by Der p1 and OVA suggests the possibility that allergens may cause or aggravate eczema through an IL-18-associated mechanism.

In conclusion, the correlation of IL-18 and IL-18BP in eczema plasma suggests an important balance between IL-18 and IL-18BP in eczema. The decrease in molar concentration ratio of plasma IL-18BP/IL-18 and allergen-induced upregulated expression of IL-18 and IL-18R in skin mast cells of the patients with eczema suggests that anti-IL-18 including IL-18BP therapy may be useful for the treatment of eczema.

## Figures and Tables

**Table 1:** Characteristics of adult subjects.

Population	Case	Age (y)	Female/male	History (y)	Onset age (y)
HC	12	25 (23–27)	8/4	0	0
Eczema	12	28.5 (19–53)	10/2	4 (0.08–15)	23 (18–51)
Cockroach (+)	2	35 (29–41)	2/0	1.5 (1-2)	33.5 (27–40)
Food (+)	2	26 (26-26)	1/1	4 (4-4)	22 (22–22)
Mite (+)	4	28 (19–35)	3/1	8.5 (0.08–15)	19.5 (18–24)
Pollen (+)	2	51.5 (50–53)	2/0	2 (2–2)	49.5 (48–51)
Small molecule (+)	1	41	1/0	5	36
Mite + pollen (+)	1	22	1/0	4	18

Median values (range) are shown. Specific allergens were examined by skin prick test. HC: healthy control.

## Abbreviations

IL: Interleukin
IL-18R: IL-18 receptor
IL-18BP: IL-18 binding protein
MFI: Mean fluorescence intensity
PBMCs: Peripheral blood mononuclear cells
TLR: Toll-like receptor
HC: Healthy control
AE: Atopic eczema
CI: Calcium ionophore.

## References

[1] Chang C., Keen C. L., Gershwin M. E. (2007). Treatment of eczema. *Clinical Reviews in Allergy & Immunology*.

[2] Fatima S., Rafiq A., Majid Z. (2015). Harlequin ichthyosis in an infant born to a father with eczema. *Journal of Tropical Pediatrics*.

[3] Novak N., Kruse S., Potreck J. (2005). Single nucleotide polymorphisms of the IL18 gene are associated with atopic eczema. *The Journal of Allergy and Clinical Immunology*.

[4] Wang D., Drenker M., Eiz‐Vesper B., Werfel T., Wittmann M. (2008). Evidence for a pathogenetic role of interleukin-18 in cutaneous lupus erythematosus. *Arthritis and Rheumatism*.

[5] Companjen A., van der Wel L., van der Fits L., Laman J., Prens E. (2004). Elevated interleukin-18 protein expression in early active and progressive plaque-type psoriatic lesions. *European Cytokine Network*.

[6] Higashi N., Gesser B., Kawana S., Thestrup-Pedersen K. (2001). Expression of IL-18 mRNA and secretion of IL-18 are reduced in monocytes from patients with atopic dermatitis. *The Journal of Allergy and Clinical Immunology*.

[7] Gutzmer R., Langer K., Mommert S., Wittmann M., Kapp A., Werfel T. (2003). Human dendritic cells express the IL-18R and are chemoattracted to IL-18. *Journal of Immunology*.

[8] Naik S. M., Cannon G., Burbach G. J. (1999). Human keratinocytes constitutively express interleukin-18 and secrete biologically active interleukin-18 after treatment with pro-inflammatory mediators and dinitrochlorobenzene. *The Journal of Investigative Dermatology*.

[9] Akira S. (2000). The role of IL-18 in innate immunity. *Current Opinion in Immunology*.

[10] Nakanishi K., Yoshimoto T., Tsutsui H., Okamura H. (2001). Interleukin-18 regulates both Th1 and Th2 responses. *Annual Review of Immunology*.

[11] Xu D., Chan W. L., Leung B. P. (1998). Selective expression and functions of interleukin 18 receptor on T helper (Th) type 1 but not Th2 cells. *The Journal of Experimental Medicine*.

[12] Sims J. E., Smith D. E. (2010). The IL-1 family: regulators of immunity. *Nature Reviews Immunology*.

[13] Wittmann M., Purwar R., Hartmann C., Gutzmer R., Werfel T. (2005). Human keratinocytes respond to interleukin-18: implication for the course of chronic inflammatory skin diseases. *The Journal of Investigative Dermatology*.

[14] Novick D., Kim S. H., Fantuzzi G., Reznikov L. L., Dinarello C. A., Rubinstein M. (1999). Interleukin-18 binding protein: a novel modulator of the Th1 cytokine response. *Immunity*.

[15] Girard C., Rech J., Brown M. (2016). Elevated serum levels of free interleukin-18 in adult-onset Still’s disease. *Rheumatology (Oxford)*.

[16] Bretterklieber A., Beham‐Schmid C., Sturm G. J. (2015). Anaphylaxis with clonal mast cells in normal looking skin - a new entity?. *Allergy*.

[17] Ando T., Matsumoto K., Namiranian S. (2013). Mast cells are required for full expression of allergen/SEB-induced skin inflammation. *The Journal of Investigative Dermatology*.

[18] Ribbing C., Engblom C., Lappalainen J. (2011). Mast cells generated from patients with atopic eczema have enhanced levels of granule mediators and an impaired Dectin-1 expression. *Allergy*.

[19] Lange L., Rietschel E., Hunzelmann N., Hartmann K. (2008). Elevated levels of tryptase in children with nummular eczema. *Allergy*.

[20] Schultz Larsen F., Hanifin J. M. (1992). Secular change in the occurrence of atopic dermatitis. *Acta Dermato-Venereologica Supplementum*.

[21] Dewan P. A., Tieu H. C., Chieng B. S. (1996). Phimosis: is circumcision necessary?. *Journal of Paediatrics and Child Health*.

[22] Jin H., He R., Oyoshi M., Geha R. S. (2009). Animal models of atopic dermatitis. *The Journal of Investigative Dermatology*.

[23] He S., Gaca M. D., Walls A. F. (1998). A role for tryptase in the activation of human mast cells: modulation of histamine release by tryptase and inhibitors of tryptase. *The Journal of Pharmacology and Experimental Therapeutics*.

[24] Vom Berg J., Vrohlings M., Haller S. (2013). Intratumoral IL-12 combined with CTLA-4 blockade elicits T cell-mediated glioma rejection. *The Journal of Experimental Medicine*.

[25] Akabane S., Matsuzaki K., Yamashita S. I. (2016). Constitutive activation of PINK1 protein leads to proteasome-mediated and non-apoptotic cell death independently of mitochondrial autophagy. *The Journal of Biological Chemistry*.

[26] He S., Zhang H., Chen H. (2010). Expression and release of IL-29 by mast cells and modulation of mast cell behavior by IL-29. *Allergy*.

[27] Zhang H., Yang X., Yang H. (2007). Modulation of mast cell proteinase-activated receptor expression and IL-4 release by IL-12. *Immunology and Cell Biology*.

[28] Novick D., Schwartsburd B., Pinkus R. (2001). A novel IL-18BP ELISA shows elevated serum IL-18BP in sepsis and extensive decrease of free IL-18. *Cytokine*.

[29] Shek L. P., Chong A. R., Soh S. E. (2010). Specific profiles of house dust mite sensitization in children with asthma and in children with eczema. *Pediatric Allergy and Immunology*.

[30] Metcalfe J. R., D’Vaz N., Makrides M. (2016). Elevated IL-5 and IL-13 responses to egg proteins predate the introduction of egg in solid foods in infants with eczema. *Clinical and Experimental Allergy*.

[31] Leach S. T., Messina I., Lemberg D. A., Novick D., Rubenstein M., Day A. S. (2008). Local and systemic interleukin-18 and interleukin-18-binding protein in children with inflammatory bowel disease. *Inflammatory Bowel Diseases*.

[32] Shao X. T., Feng L., Gu L. J. (2009). Expression of interleukin-18, IL-18BP, and IL-18R in serum, synovial fluid, and synovial tissue in patients with rheumatoid arthritis. *Clinical and Experimental Medicine*.

[33] Migliorini P., Anzilotti C., Pratesi F. (2010). Serum and urinary levels of IL-18 and its inhibitor IL-18BP in systemic lupus erythematosus. *European Cytokine Network*.

[34] Arend W. P., Palmer G., Gabay C. (2008). IL-1, IL-18, and IL-33 families of cytokines. *Immunological Reviews*.

[35] Wittmann M., Macdonald A., Renne J. (2009). IL-18 and skin inflammation. *Autoimmunity Reviews*.

[36] Yoshimoto T., Mizutani H., Tsutsui H. (2000). IL-18 induction of IgE: dependence on CD4+ T cells, IL-4 and STAT6. *Nature Immunology*.

[37] Yoshimoto T., Tsutsui H., Tominaga K. (1999). IL-18, although antiallergic when administered with IL-12, stimulates IL-4 and histamine release by basophils. *Proceedings of the National Academy of Sciences of the United States of America*.

[38] Hoshino T., Yagita H., Ortaldo J. R., Wiltrout R. H., Young H. A. (2000). In vivo administration of IL-18 can induce IgE production through Th2 cytokine induction and up-regulation of CD40 ligand (CD154) expression on CD4+ T cells. *European Journal of Immunology*.

[39] Konishi H., Tsutsui H., Murakami T. (2002). IL-18 contributes to the spontaneous development of atopic dermatitis-like inflammatory skin lesion independently of IgE/stat6 under specific pathogen-free conditions. *Proceedings of the National Academy of Sciences of the United States of America*.

[40] Lind S. M., Kuylenstierna C., Moll M. (2009). IL-18 skews the invariant NKT-cell population via autoreactive activation in atopic eczema. *European Journal of Immunology*.

[41] Zhan M., Zheng W., Jiang Q. (2016). Upregulated expression of substance P (SP) and NK1R in eczema and SP induced mast cell accumulation. *Cell Biology and Toxicology*.

[42] He S., Gaça M. D., AR M. E., Walls A. F. (1999). Inhibitors of chymase as mast cell-stabilizing agents: contribution of chymase in the activation of human mast cells. *The Journal of Pharmacology and Experimental Therapeutics*.

[43] Tsutsui H., Mizutani H., Nakanishi K. (2011). Contribution of interleukin 18 to the development of infection-associated atopic dermatitis. *Current Problems in Dermatology*.

[44] Wittmann M., Doble R., Bachmann M., Pfeilschifter J., Werfel T., Mühl H. (2012). IL-27 regulates IL-18 binding protein in skin resident cells. *PLoS One*.

[45] Kannan Y., Yu J., Raices R. M. (2011). IkappaBzeta augments IL-12- and IL-18-mediated IFN-gamma production in human NK cells. *Blood*.

[46] Paulukat J., Bosmann M., Nold M. (2001). Expression and release of IL-18 binding protein in response to IFN-gamma. *Journal of Immunology*.

[47] Veenstra K. G., Jonak Z. L., Trulli S., Gollob J. A. (2002). IL-12 induces monocyte IL-18 binding protein expression via IFN-gamma. *Journal of Immunology*.

[48] Sellge G., Barkowsky M., Kramer S. (2014). Interferon-gamma regulates growth and controls Fcgamma receptor expression and activation in human intestinal mast cells. *BMC Immunology*.

